# Advancing research opportunities and promoting pathways in graduate education: a systemic approach to BUILD training at California State University, Long Beach (CSULB)

**DOI:** 10.1186/s12919-017-0088-3

**Published:** 2017-12-04

**Authors:** Guido G. Urizar, Laura Henriques, Chi-Ah Chun, Paul Buonora, Kim-Phuong L. Vu, Gino Galvez, Laura Kingsford

**Affiliations:** 1 Department of Psychology, California State University, Long Beach, CA 90840 USA; 2 Department of Science Education, California State University, Long Beach, CA 90840 USA; 3 Department of Chemistry & Biochemistry, California State University, Long Beach, CA 90840 USA; 4 College of Natural Sciences & Mathematics, California State University, Long Beach, CA 90840 USA

## Abstract

**Background and purpose:**

First-generation college graduates, racial and ethnic minorities, people with disabilities, and those from disadvantaged backgrounds are gravely underrepresented in the health research workforce representing behavioral health sciences and biomedical sciences and engineering (BHS/BSE). Furthermore, relative to their peers, very few students from these underrepresented groups (URGs) earn scientific bachelor’s degrees with even fewer earning doctorate degrees. Therefore, programs that engage and retain URGs in health-related research careers early on in their career path are imperative to promote the diversity of well-trained research scientists who have the ability to address the nation’s complex health challenges in an interdisciplinary way. The purpose of this paper is to describe the challenges, lessons learned, and sustainability of implementing a large-scale, multidisciplinary research infrastructure at California State University, Long Beach (CSULB) – a minority-serving institution – through federal funding received by the National Institutes of Health (NIH) Building Infrastructure Leading to Diversity (BUILD) Initiative.

**Program and key highlights:**

The CSULB BUILD initiative consists of developing a research infrastructure designed to engage and retain URGs on the research career path by providing them with the research training and skills needed to make them highly competitive for doctoral programs and entry into the research workforce. This initiative unites many research disciplines using basic, applied, and translational approaches to offer insights and develop technologies addressing prominent community and national health issues from a multidisciplinary perspective. Additionally, this initiative brings together local (e.g., high school, community college, doctoral research institutions) and national (e.g., National Research Mentoring Network) collaborative partners to alter how we identify, develop, and implement resources to enhance student and faculty research. Finally, this initiative establishes a student research training program that engages URGs earlier in their academic development, is larger and multidisciplinary in scope, and is responsive to the life contexts and promotes the cultural capital that URGs bring to their career path.

**Implications:**

Although there have been many challenges to planning for and developing CSULB BUILD’s large-scale, multidisciplinary research infrastructure, there have been many lessons learned in the process that could aid other campuses in the development and sustainability of similar research programs.

**Electronic supplementary material:**

The online version of this article (10.1186/s12919-017-0088-3) contains supplementary material, which is available to authorized users.

## Background

As is well documented, first generation-educated racial and ethnic minorities (e.g., Latino, African American, Pacific Islander, Native Alaskans and Hawaiians, and Native American), people with disabilities, and those from disadvantaged backgrounds are gravely underrepresented in the health-related research disciplines and workforce representing behavioral health sciences and biomedical sciences and engineering (BHS/BSE) [1, 2]. National data from the U.S. shows that relative to their peers, comparable percentages of students from these underrepresented groups (URGs) show strong interest in BHS/BSE and in pursuing scientific majors [3]. However, URGs represent only 14% of earned scientific bachelor’s degrees, compared with 81% for their non-URG peers (i.e., White & Asian American) [4]. This pattern continues in graduate school with only 6% of BHS/BSE doctorates awarded to URGs compared to 74% for non-URGS [1, 2]. Therefore, programs that engage and retain URGs in health-related research careers are imperative to promote the diversity of well-trained research scientists who have the necessary research skills to be leaders in their respective fields, as well as the ability to address the nation’s complex health challenges in an interdisciplinary way, to ensure that the next generation of BHS/BSE researchers reflects the diversity of the overall U.S. population.

The purpose of this paper is to describe the challenges, lessons learned, and sustainability of implementing a large-scale, multidisciplinary research infrastructure at California State University, Long Beach (CSULB) – a minority-serving institution – through federal funding received by the National Institutes of Health (NIH) Building Infrastructure Leading to Diversity (BUILD) Initiative. The CSULB BUILD initiative is one of ten BUILD sites in the U.S. and is designed to engage and retain URGs on the research career path by providing them with the research training and skills needed to make them highly competitive for doctoral programs and entry into the research workforce. Specifically, this paper describes:

1. Our campus’ institutional background, as well as the development of a diversity consortium and implementation of a campus-wide needs assessment that helped to inform and guide the implementation of the CSULB BUILD Initiative;
2. How we are developing a campus-wide culture of multidisciplinary collaboration to address different research infrastructure needs and strengthen the quality of research being conducted;
3. The challenges associated with transforming student research training from an extra-curricular activity to a curricular one in order to engage students in research early and often in their college experience;
4. The implementation of research infrastructure, resources, and workshops designed to support faculty research and mentorship of students;
5. The challenges associated with implementing a large-scale, multidisciplinary student research training program to engage and retain students in the research career path;
6. The critical role that our community college and doctoral research collaborative partners have had in strengthening the CSULB BUILD Initiative; and
7. Efforts and challenges to institutionalizing and sustaining program components for students, faculty, and other key stakeholders long-term.

### Institutional background

CSULB, with more than 37,000 students, has been a Hispanic Serving Institution (HSI) since 2005 and an Asian American and Native American Pacific Islander-Serving Institution (AANAPISI) since 2011, CSULB is located within one of our nation’s most diverse cities [5, 6], providing an ideal environment for the development of student research scientists, particularly those from first generation-educated, underrepresented backgrounds. Based on self-reported data, the largest ethnic group on campus is Hispanic, who in fall 2016 represented 45.6% of total undergraduates. African Americans, Pacific Islanders, and Native Americans represented an additional 4.7% of undergraduates. Over half of CSULB students are first generation-educated, lower-income, and Pell Grant eligible. In *Diverse Issues in Higher Education* (2015) [7]*,* CSULB was ranked 6th in the nation among universities conferring bachelor’s degrees in 2013–14 to URGs, including 6th nationally among those conferring bachelor’s degrees to Hispanics and 21st in bachelor’s degrees to Native Americans. Overall, CSULB’s campus-wide graduation percentages for URGs mirror their enrollment percentages, suggesting a positive and high retention and completion profile. In departments representing BHS/BSE in 2012, the graduation rates of URGs were higher than the national average (~34% at CSULB vs. 14% nationally) [8]. Just as CSULB has a long history of serving a diverse student population, it also has a long history of supporting student research through externally funded training programs. As seen in Additional file 1, with funding from NIH, NSF, USDA and others, CSULB has supported and trained more than 1000 students over the past three decades. As these data demonstrate, CSULB has been a leader in preparing students from traditionally underserved communities in obtaining higher education.

### Planning for CSULB BUILD (the AHORA initiative)

Despite CSULB’s long history of student research training, many of these earlier programs focused on a smaller cohort of students (4 to 12) representing majors within a single college. These research training programs have traditionally been run within four colleges – College of Liberal Arts (CLA), College of Health and Human Services (CHHS), College of Natural Sciences and Mathematics (CNSM), and the College of Engineering (COE). However, they often worked in isolation from one another, used a single principal investigator (PI) system with little collaboration across disciplines, and were not institutionalized when funding ended. Given the limitations of these earlier programs, three CSULB faculty members (representing CLA, CHHS, and CNSM) worked with campus administrators to develop a three PI-system and apply for a NIMHD BUILD planning grant, which was funded in fall 2013. The primary objective of this grant was to develop the AHORA (Alliance for Health Opportunities Research Advancement) initiative [9], which focused on assembling a consortium of URG research partners, doctoral program directors, faculty, academic advisors, and students (both current and alumni in PhD programs). This consortium helped to assess student research training programs and institutional resources at CSULB. They also participated in focus groups, one-on-one interviews, and a one-day conference to share their experiences of the barriers that URGs face as they go through the doctoral pipeline. This data was analyzed and written into a best practices report for campus administrators and NIH that resulted in the emergence of six themes to inform strategic planning for engaging and retaining URGs in BHS/BSE research including: 1) perceptions of diversity and how it adds to the educational experience, 2) experiences of discrimination in academia, 3) effective mentorship, 4) barriers to academic success, 5) students’ cultural capital and assets, and 6) successful program components [9]. Collectively, results from the AHORA initiative demonstrate that *operating student research and mentorship programs in isolation does not take advantage of the diverse on- and off-campus resources, student peer interaction, and common initiatives for promoting graduate education that a unifying institutional infrastructure would provide.* The standard approach to promoting graduate education has been a “program-specific” model wherein on-campus student research and mentorship programs often work separately and develop programmatic curriculum and resources to support their small cohort of students towards doctorate programs. This approach constrains programmatic ability for resource sharing, building a strong and visible campus presence for URGs, and creating support networks. Additionally, it does not provide alternate pathways for students interested in changing majors within the BHS/BSE fields. Consequently, students often lack awareness of available mentorship and resources to engage in research. These AHORA results were instrumental in helping to inform the design and implementation of the CSULB BUILD Initiative and were obtained through qualitative and quantitative assessments (across different stakeholders) that could be used by institutions that are looking to explore and assess their own infrastructure needs to identify resources needed to implement campus-wide research training programs focused on engaging and retaining URGs in research career paths.

### Challenges in developing a culture of multidisciplinary collaboration

To challenge the standard “program-specific” model of promoting graduate education, the CSULB BUILD initiative seeks to *shift the institutional research and mentorship culture on the CSULB campus* by unifying existing on-campus partners, our community college and local research university partners, and our national network of doctoral research programs to fortify URG research training and career development. This starts at the leadership level, with our principal investigators, program directors, co-directors, and members of our advisory boards and steering committee representing several disciplines of health research. This formative work distinctively *builds a unique alliance between the different health research disciplines at CSULB in order to bring together often disparate areas of science*, with the common goal of increasing the number of URGs in the health research work force. We expect this alliance to lead to collaborations that offer a breadth of multidisciplinary research and mentorship opportunities for URGs.

The single biggest challenge we have faced in developing the CSULB BUILD program comes from the fact that this is a campus-wide initiative. Unlike earlier programs which focused on students from a smaller number of majors within a single college, BUILD requires coordination, leadership, and buy-in across multiple colleges and majors. The infrastructure, administrative issues, and learning of different academic and research cultures required for crossing borders between departments and academic units cannot be understated. Not only do faculty and administrators need to work across disciplines, every activity we do with students needs to be translated to the language and culture of the students’ different academic discipline. An activity as seemingly straightforward as teaching students how to write a curriculum vitae (CV), for example, quickly becomes complex as the style and content of CVs in different fields varies. Another example comes from the way BUILD programs have been branded by NIH to promote “biomedical” research. This terminology was very problematic during the first couple of years of developing the BUILD program because the label of biomedical research does not resonate with the majority of health-research scientists that represent the wide array of research in the behavioral, clinical, and social sciences. This affected how BUILD was initially branded and viewed by key stakeholders (i.e., students, academic advisors, faculty, administrators, community college and research partners) on and off our campus, it affected our outreach efforts to recruit students and faculty, and it affected how we framed our programmatic activities and opportunities for career development. Specifically, our stakeholders viewed BUILD as a program that catered to CNSM students and faculty in the research areas of biology and chemistry. This required several discussions within our leadership team and consultation with our advisory boards (which include our community college and doctoral research partners) and steering committee to get rid of the term “biomedical” from our BUILD website, in all of our promotional materials, and most importantly, in the way we design and deliver programmatic activities to BUILD students participating in our learning communities and faculty participating in our career development workshops (both are described in more detail below). This unified approach has helped our leadership team to champion for students that NIH may not traditionally view as conducting “biomedical” research because they have majors in political science or religious studies, but who are clearly conducting innovative research with faculty mentors examining health disparities and public health issues in family health and behavioral neuroscience and who have a strong commitment to pursue graduate studies in health-related fields. In essence, shifting the institutional research culture has involved educating ourselves and NIH with where and how health research is being studied in different departments and majors on our campus in order for us to be more inclusive and innovative in supporting the research that is being conducted and that is more typical of that seen in federally-funded, multidisciplinary research institutions around the world that use more holistic frameworks, such as the “biopsychosocial model” of health, to address prominent health issues [10].

BUILD has great potential and promise, but it is no small task in creating the procedures and mechanisms to get a program this large started and thriving. Without a unified and organized approach to working with the entire campus, we cannot transform the university in ways which will be lasting and maximize student success. While the development of a supporting infrastructure was part of our project goals, we underestimated the time and effort required for developing the non-tangible aspects of campus infrastructure, which are critical for the success of this initiative. *While BUILD capitalizes on lessons learned from earlier programs in regards to training students, BUILD also aims to transform the culture of the campus so that significant features of BUILD remain after funding ends.*

### Challenges in institutionalizing student research curriculum

To enhance student research training on campus, CSULB BUILD focused on developing research-infused curriculum across colleges. Results from our AHORA initiative and its assessment of student research training showed that policies and practices supporting curricular research experiences varied widely between departments and colleges. Some departments had career exploration-focused courses, and some had courses designed to provide specific skills training that would support an aspiring researcher. The assessment also noted that a large body of research skill development and preparation for graduate study was offered through grant-funded student research training programs, such as the MARC U*STAR, LSAMP, HSI STEM and RISE (see Additional file 1). While these grant-funded activities are open to the entire campus, ensuring student awareness and scheduling them for a largely commuter and working student population limited participation. Our goal in support of the mission to enhance interest and preparation for entry into research careers is to move research training from an extra-curricular activity, often available to a limited number of participants, to a curricular one, where research-infused courses are available to all students across colleges and count towards degree requirements for graduation. An advantage of a campus-wide curriculum for some departments is the opportunity to provide research skills training without having to establish new courses where the numbers of participating students might initially be small. Campus-wide courses effectively pool students, and as demand increases, individual disciplines or small groups of related disciplines could justify creating their own sections of courses.

In our initial development stage, we sought feedback from representatives of each of the four colleges participating in the BUILD initiative (CLA, CHHS, CNSM, COE) and asked them to list individual skill sets needed to be successful in graduate study and beyond (see Additional file 2). Results were then organized around related topics resulting in the development of five courses. The objective of this course development was to ensure we had a true, coherent curriculum with articulated learning outcomes rather than a collection of isolated courses.

The first course was a *Career Exploration* course for freshmen. The class objectives were to engage entry level students to (a) explore the full range of career options, (b) become aware of the cultural and community assets they can bring to careers, and (c) cultivate an identity as a scientist, all with an emphasis on possible research careers. Course activities focus on scientist identity and mindset factors influencing career selection based on published evidence-based interventions. Having acquired a clearer vision of a rewarding career path, students create individual development plans (IDPs) they can use and refer to as they move through their undergraduate education. We plan to share the course with partners at the community colleges so that this course can be offered on their campuses. The course can also serve as a mechanism for recruiting new students into BUILD and BHS/BSE research careers.

The remaining four courses, while open to all students, are required of BUILD student trainees and are designed to create a research-infused curriculum. The curriculum begins with *Introduction to Research Methods*, a sophomore course introducing principles of experimentation, hypotheses formulation and testing, measurement, naturalistic observation, correlational studies, analysis, and reporting. Two versions of this course were developed, one each for the BHS and BSE disciplines, in recognition of differences in needed disciplinary skills. The other sophomore course is *Introduction to Health Disparities*, an interdisciplinary course that explores socioeconomic, biological, environmental, and institutional factors associated with health issues and disease. Students learn about research and interventions that affect positive health outcomes and access for underserved, underrepresented, and diverse populations.

At the junior-level, we developed a *Scientific Research Communication* course that helps build proficiency in oral and written communication and intensive practice in writing, editing, and evaluating scientific reports, with specific reference to discipline-specific methodologies as related to scientific inquiry and research. The last course, *Advanced Research Design & Methods*, engages students in hypothesis testing, experimental design, methodological and technical procedures for experimentation, identifying funding sources for their research (NIH and other sources), and grant writing. Like *the Introduction to Research Methods* course, we created BHS and BSE versions of this course to address the differences in emphasis and content [*please see* Additional file 2 *for an overview of student learning goals across the courses*].

Creating the courses does not ensure their institutionalization, so planning to avoid pitfalls was developed. So that the curriculum was not perceived as applying only to BUILD trainees, the curriculum was tagged as the “CSULB Research Curriculum.” The courses were designed to fit into the university’s general education (GE) curriculum to reduce the potential impact on the students’ course unit total and time to completion. The courses are being presented to individual departments for consideration as potential electives within existing degree programs. With most CSULB majors at the allowed 120-unit maximum, adding courses as elective options should not extend the students’ time to degree completion.

The process of executing the planned development has met with challenges. As a campus-wide curriculum, the courses are interdisciplinary and cross-listed between colleges. Without a clear “ownership” of a single academic unit identifying available faculty and questions of who schedules rooms and pays instructors has required a team effort across four colleges. Recognizing that this effort will be difficult to sustain, we are looking into the creation of a research course prefix and moving the courses to a central office within the university. The Office of Research and Sponsored Programs (ORSP) has taken a leadership role in meetings with key campus administrators and stakeholders to begin the process of institutionalization of these courses.

### Fortifying the research infrastructure

In addition to enhancing our research curriculum for students, findings from the AHORA initiative revealed that the CSULB campus has not adequately integrated its BHS/BSE research infrastructure. Specifically, there is very limited sharing and access to research resources, poor interdisciplinary communication and collaboration across colleges, and a lack of consistency in the respective colleges’ “research cultures” and administrative support. Additionally, faculty perceive that their time and effort with student mentorship is undervalued. These findings have led to substantive institutional support from CSULB’s administrators to support faculty, as well as enhance and sustain student research training and mentorship activities. The CSULB BUILD initiative has established a strategic plan for research infrastructure intended to leverage and integrate existing strengths, reduce costly redundancies through resource sharing, and facilitate interdisciplinary and multidisciplinary collaborations. Coordination among large numbers of departments in four different colleges is a challenging task. To achieve these goals, CSULB BUILD devoted monetary resources to (a) establish or equip shared research space in support of CSULB’s research infrastructure, (b) fund competitive awards in support of individual and collaborative faculty research, and (c) fund or sponsor technical training workshops for faculty and students that are open to participants from any discipline. In addition, BUILD committed resources to faculty training and support in the areas of cultural competency and sharing best practices in mentoring and teaching, as part of a BUILD mentoring community that includes participation from faculty across all four colleges. By adding diversity in terms of disciplines, faculty are able to more freely communicate about specific issues and obtain multiple perspectives from colleagues across campus for whom they would otherwise have no interaction.

An important piece of this initiative was investing in and fortifying CSULB’s research infrastructure across the four colleges participating in BUILD (CHHS, CLA, CNSM, and COE). The challenging task of determining how to allocate these resources was met by addressing the unique and greatest needs for each college. For CHHS, a new Interdisciplinary Health Research Laboratory was established to help provide faculty with research space to conduct different types of research studies, from interviews and focus groups to health-related interventions. For CLA, funds provided by BUILD were supplemented by a 2.9 million dollar investment from the university to support the major construction of three wet labs in the Psychology Building to increase research and student training in the area of behavioral neuroscience. To extend CNSM’s research capability, a state-of-the-art cell-sorter was purchased as a shared resource for the college. For COE, BUILD partially supported the renovation of a research facility, as well as a high fidelity 3-D printer and scanning software, to support the research and student training activities for the newly established BS degree program in biomedical engineering. The Provost also committed university space for a *Research Success and Student Engagement Center* which houses the BUILD headquarters and provides the infrastructure needed to connect students and faculty to research resources and career development opportunities on campus, as well as those of community college partners and doctoral research partners. Despite the challenges common to establishing new research infrastructure, these investments are anticipated to have far-reaching impact in terms of research capacity for the campus.

#### Faculty research and mentoring development

In addition to fortifying our campus’ research infrastructure, BUILD faculty’s research and mentoring activities are supported in two other ways. Small grants support faculty and student research, while a series of workshops and professional learning opportunities support faculty’s development as mentors and instructors. CSULB BUILD offers several competitive awards for faculty to strengthen and enhance their individual and collaborative research programs as a form of capacity building for the University. The first type of award is the Research Stimulation Grant (RSG; individual research program) and Collaborative Research Stimulation Grant (CRSG, in collaboration with a R01-level PI to enhance the CSULB faculty’s research program), which provide faculty PIs with seed money for pilot projects. Data collected from these projects are intended to help the PIs meet the requirement of submitting a grant proposal to an external funding source in order to increase their competitiveness for external funding. The second award is the Small/Midsize Equipment and Computer Award. This award allows faculty to equip their own labs with computers and/or smaller equipment (between $5000 and $24,999) to support BUILD student-mentored research. In addition to these awards, BUILD also provides funding and support for technical training workshops to enhance the skills, knowledge, and abilities of our faculty and students to engage in a wide variety of research topics, instruments, methodologies, and statistical analyses representing various areas of BHS/BSE research.

#### BUILD mentoring community

In addition to grant support and technical training workshops, faculty also receive peer support for their mentoring activities with BUILD student trainees. In collaboration with the National Research Mentoring Network (NRMN), we developed and implemented a BUILD mentoring community (BMC) program to promote faculty skills in active learning methods and research-emphasized pedagogy when mentoring students in research and classroom settings. This 10-week hybrid program consists of two face-to-face meetings with other faculty and an online forum which allows BMC members to read, discuss, and share personal expertise in mentoring philosophy, aligning expectations of mentors with mentees, fostering student independence, creating a mentoring plan, as well as identifying ways to promote effective communication and students’ cultural capital [11, 12]. We also provide a series of multicultural workshops that supplement the BMC and cover topics that include examining stereotypical assumptions about language and ethnic minority students in learning situations and the effects that these assumptions have on faculty’s perception of students’ academic preparedness and success [13, 14]. The content and format of the BMC and these multicultural workshops were formulated in response to results from our AHORA initiative, which demonstrated that standard approaches to mentoring students in research include environments that promote a student deficit model (focused on correcting student deficiencies) and are often void of understanding the resilience and cultural strengths, as well as the obstacles that URGs have overcome to enroll in college and engage in research [9]. Therefore, having faculty who are culturally responsive and supportive of students is an important factor for student success. A second, and important, component of the BMC is a second semester project where faculty pilot test and assess refinements in their mentoring skills in order to put new ideas into practice and evaluate their effectiveness in improving students’ experiences with research training and professional development. After completing the project, faculty write a short summary of their findings and share what they have learned with their peers. A common challenge in implementing these types of programs is getting faculty buy-in to participate in these professional development activities. To support BUILD faculty mentors who complete the BMC and who attend one of our multicultural workshops, we provide them with research supply money ($1800) to support their collaborative work with BUILD students. Additionally, we provide faculty with a $1000 stipend for completing the BMC’s second semester project. In collaboration with NRMN, our faculty receive a certificate of completion of CSULB-NRMN training at the end of the program. Faculty are encouraged to include this documentation of program completion in their files for retention, tenure, or promotion (RTP), as well as include this information on their NIH biosketches. We are currently in discussions with campus administrators to see how participation in these programs can be promoted, sustained, and valued by RTP committees when faculty are reviewed for retention, tenure, or promotion.

### Implementing a multidisciplinary student research training program

**Fig. 1:**
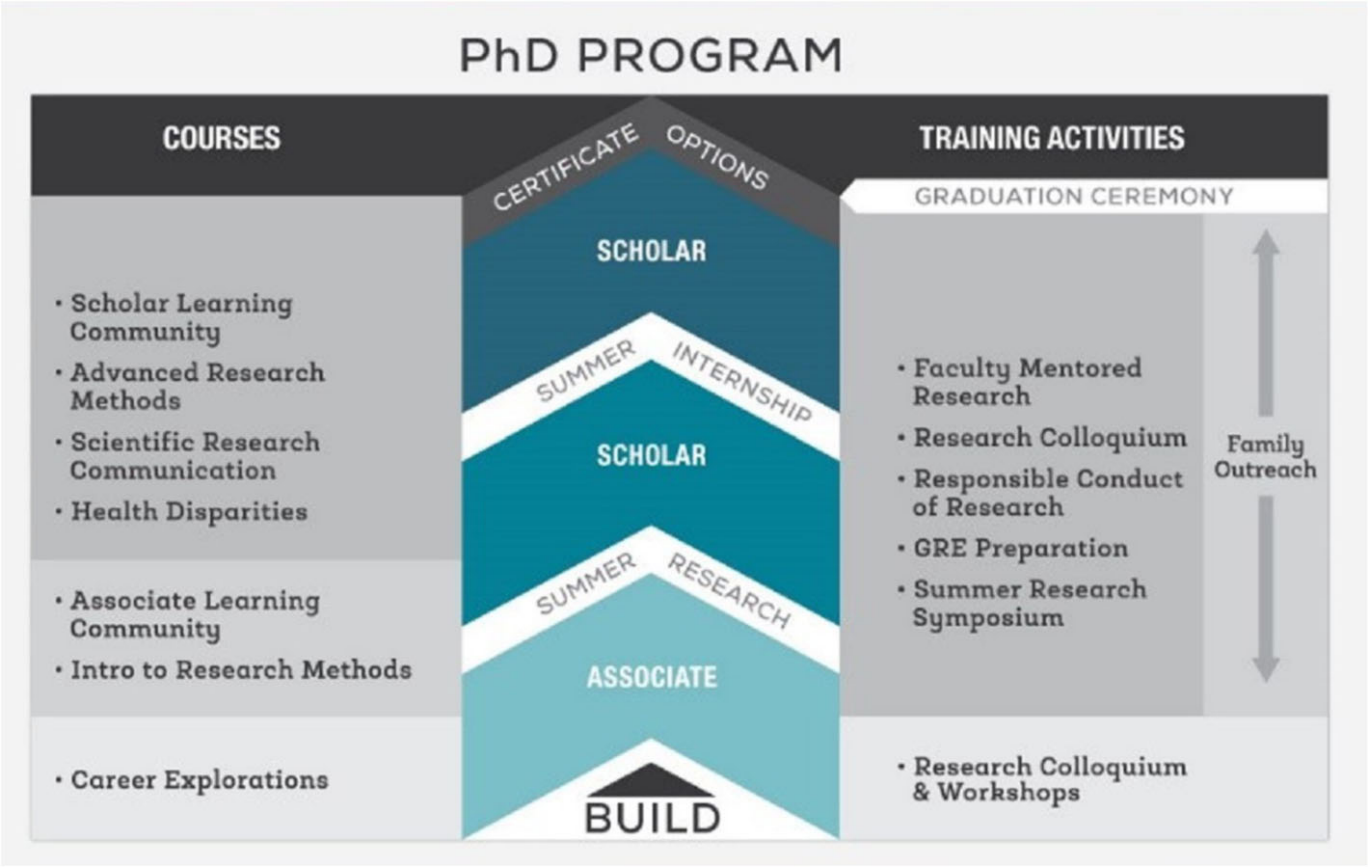
Overview of the CSULB Student Training Program

Several factors can serve as barriers to URGs engaging in research. These include academic environments that lack institutional diversity (few faculty and student role models, few resources allocated to diversity initiatives), which can lead to URGs feeling isolated and showing lower levels of commitment in proactively navigating and pursuing training opportunities for graduate school [15, 16]. Many URGs are also the first in their family to attend college and, therefore, experience a lack of family understanding and support for their aspirations to attend graduate school (which can occur for parents who have received little to no information regarding career options for URGs who pursue research paths) [14, 18]. Finally, many students face socioeconomic adversity that require them to contribute to their family’s economic survival, making it difficult to balance work and school and limiting their engagement in on-campus research activities [17, 18]. As such, innovative programs honoring URGs’ life contexts are critical to engage students in BHS/BSE research career paths. The CSULB BUILD student research training program was designed to address several of these barriers to higher education by cultivating students’ culturally congruent science identity and sense of belonging in the research environment via continuous research exposure, academic support, supplemental instruction, and multi-tiered mentoring that integrates family support systems and students’ cultural capital [11, 19, 20]. In addition, BUILD activities use active learning approaches and project-based learning that is meaningful to the students’ values and relevant to their respective communities [21, 22]. Students are recruited at the freshmen and sophomore levels to support early engagement in BHS/BSE research. Freshmen with strong science interest and academic potential are recruited to participate in our nine-month BUILD Associates Program beginning in their sophomore year. Sophomores are subsequently recruited to participate in the BUILD Scholars Program, which is a two-year program (i.e., junior and senior years) that includes Scholars participating in an 8-week Summer Research Training Program in their first year in the program and in an off-campus Summer Research Internship at a doctoral-granting institution in their second year. As illustrated in Fig. 1, students have the opportunity to move through Lower and Upper Division training curricula that emphasize continuous research training from the sophomore to senior year. Altogether, our current group of sophomores, juniors, and continuing seniors equates to 110 active BUILD student trainees for the 2016–2017 academic year, representing the largest student cohort of the 10 BUILD sites.

The key components of the BUILD Associates and Scholars programs are a year-round Learning Community seminar and faculty-mentored research training, as well as participation in the BUILD Research Colloquium, Graduate Record Examination (GRE) study pods, and family connection events. These components are supplemented by substantial financial (i.e., tuition, stipend, funds for research supplies and conference travel) and academic support (e.g., priority registration, in-house writing specialist, and in-house education counselor). The training goals of the BUILD Associates and Scholars programs are for students to: (a) acquire basic and advanced research methods and statistical skills via required coursework and mentored research experiences, (b) learn about the current directions and cutting-edge BHS/BSE research, (c) develop and conduct an independent research project, (d) learn to disseminate research findings, and (e) develop a culturally congruent science identity.

#### Learning community seminar

The Learning Community (LC) seminar utilizes a student cohort model to create a formal support system for BUILD trainees and provides them with the opportunity to apply research concepts learned in the classroom and research setting, develop research and professional skills, and cultivate personal and cultural assets in a supportive environment that is supplemented by curriculum, workshops, and colloquia. The LC seminars are run separately for each level of the program. Given the large scale of the CSULB BUILD program, multiple sections are offered and facilitated by LC Graduate Assistants (near-peer mentors). This multi-tiered, near-peer mentoring model allows our trainees to receive additional support and advice to allow them to identify what works in supporting their own academic success. This model has been shown to promote student belonging and identity development better than single mentor models [11, 19] and is a key component of our student training program. Another major emphasis of the learning community is to involve students’ families in BUILD events such as our Summer Research Symposium, where students present a research poster on their work to faculty mentors, families, and friends. These events aim to increase family support and understanding of the career paths and demands that our trainees experience, as well as offer a unique and novel method to enhance students’ cultural congruence of their scientific identity [20]. The integration of cultural factors (e.g., developing and strengthening growth mindset, stereotype threat, attribution error) in building a relevant science identity for URGs has the added benefit of utilizing cultural capital to enhance student outcomes. The *Student Cultural Capital Model*, adapted and developed through the National Center of La Raza/CSULB Center for Latino Community Health, Evaluation & Leadership Training [11], creates an environment that promotes understanding of resilience, cultural strengths, and barriers experienced by URGs. Applying this model acknowledges the assets and strengths (i.e., cultural capital) that URGs bring to academic settings and their research career paths. To promote student awareness of their cultural capital, the LC includes activities that facilitate Scholars writing their personal statements, forcing them to think about *why* they are pursuing graduate education and *what* they would like to accomplish in and beyond graduate school, thereby helping them to articulate their culturally congruent scientist identity.

#### Research training

Upon entering the program, BUILD Associates and Scholars are paired with a faculty mentor, a training director, and a graduate assistant to foster a multi-tiered mentoring experience. Trainees are placed in research-active labs (as measured by publications, procurement of external funding, presentations at national conferences), and expected to participate in research projects, spending 10–15 h per week in the lab during the academic year. Associate trainees are expected to produce a research proposal for their independent research project that they would complete as Scholars by the end of the academic year, while Scholars are expected to design and complete an independent research project by the end of their two-year program under the guidance of their faculty mentor. In addition to these faculty-mentored research experiences, BUILD Associates and Scholars learn cutting edge BHS/BSE research by attending the monthly BUILD Research Colloquia series (speakers often consist of research faculty from our research partner institutions). By the end of the multi-year BUILD training, our Scholars will have made a minimum of seven paper presentations and four poster presentations in diverse settings, ranging from the relatively non-threatening and intimate (our LC seminars) to the professional and challenging (Annual Biomedical Research Conference for Minority Students - ABCRMS, Society for Advancement of Chicanos/Hispanics and Native Americans in Science- SACNAS and other national discipline-specific conferences). Trainees are also encouraged to present at on-campus research fairs and competitions and the CSU Research Competition.

#### Barriers and challenges to student research training

When developing the proposal for our training program, we were primarily focused on building on the rich cultural assets of our students. However, in implementation of “scaling up” we learned in a rather painful way how naively optimistic we were. Training activities that we had developed and honed with typical groups of 6–12 students in prior research training programs did not easily translate to providing training to over 100 trainees ranging from sophomores to seniors from over twenty majors across four colleges (see Table 1). In addition to the 110 active BUILD student trainees in the program, there are more than 80 faculty mentors that work with them, 6 BUILD training directors, two staff members dedicated to student training, and 18 graduate assistants (GAs). One quickly senses the enormity of the program and the complexity that its size and diversity in research disciplines bring to the table. Finding a common language in health research even among the training directors was a challenge. Identifying and articulating training priorities that all BUILD disciplines could agree on was yet another. Simple assignments such as “how to read a primary journal article” was no longer simple to implement. Almost every training activity involved extensive planning and coordination among faculty, training directors, GAs and trainees whether it be something as simple as providing feedback on a draft of a CV to something massive as taking 40 plus trainees to ABRCMS or SACNAS. Therefore, we created a Learning Community Specialist position who assists the 6 training directors with the development, adaptation, and manualization of the Learning Community activities and curriculum. The Learning Community Specialist is also in charge of training and supervising the 16 GAs. Vigilant monitoring of trainee’s academic performance and engagement becomes essential to making sure that we have a pulse on how they are doing. To address these challenges, we developed multi-level mechanisms such as weekly student activity logs, trimester trainee evaluation by faculty mentors and GAs, biannual Individual Development Plans, and individual meetings with GAs and BUILD training faculty to ensure that problems are caught early and timely support of students is provided. Finally, communications with mentors regarding upcoming training activities or evaluations are made via monthly e-newsletters.

**Table 1:** 2016-2017 BUILD Participation by College

BUILD Student Status	CHHS	COE	CLA	CNSM	Totals
BUILD Associates	1	7	5	16	29
BUILD Year 1 Scholars	4	12	15	10	41
BUILD Year 2 Scholars	7	7	9	17	40
Totals	12	26	29	43	110

*BUILD* Building Infrastructure Leading to Diversity, *CHHS* College of Health & Human Services, *COE* College of Engineering, *CLA* College of Liberal Arts, *CNSM* College of Natural Sciences & Mathematics

### Collaborative role of pipeline and research partners

A key component of the CSULB BUILD Initiative has been our pipeline and research partnerships with local community colleges and doctoral research universities, respectively. As part of BUILD, we established an external advisory board that includes several members from our pipeline and research partners in order to obtain their expertise and feedback on all BUILD activities, particularly those related to challenges in developing a culture of multidisciplinary collaboration, in institutionalizing student research curriculum, in fortifying CSULB’s research infrastructure, and in implementing a large-scale student research training program that infuses culturally-responsive pedagogies. We have applied their feedback in branding BUILD to a wider audience of students and faculty through infrastructure support for “health-related research”. Furthermore, they have been instrumental in sharing resources to support events and workshops for community college students, CSULB BUILD student trainees, and faculty from CSULB and our research partners (described in more detail below). The synergy for these partnerships stem from previously established relationships and contacts that CSULB faculty had with key stakeholders at these institutions (through research collaborations, involvement in previous student research training programs) which were fortified with our pipeline and research partners’ participation in our AHORA diversity consortium and needs assessment (conducted in preparation of CSULB BUILD) to identify priority areas for collaboration that would mutually benefit the diversity initiatives for campus administrators at CSULB and those at our partner institutions. Examples of these activities are described below.

#### Pipeline partnerships

Our pipeline partners for CSULB BUILD consist of four community colleges (CC) and a local K-12 school district that collaborate with student outreach of transfer students and freshmen to CSULB. Results from our AHORA Initiative show that transfer students (and freshmen) often feel isolated when joining a new campus, which presents an opportunity to engage these students early on through involvement in research to increase their sense of belonging, help them focus on a career trajectory, and increase graduation rates. Many students at our local community colleges engage in research with their professors through research methods classes (or other research-related courses) where they are introduced to research experiments, data collection, statistical analyses, and research writing. However, these students are often unaware of how to get involved in research when they transfer to a 4-year college, or attempt to do so much too late in the process. CSULB BUILD provides a unique opportunity to join forces with our pipeline partners to share opportunities for continued research training at CSULB, while supporting the student initiatives that our partners have on their campuses. Mutual student outreach efforts have consisted of co-hosting face-to-face BUILD information sessions and in-class announcements on their campus, sending targeted information emails to transfer students; housing display cases on the CC campuses which showcase BUILD and recent campus alumni currently in the BUILD program; posting fliers and posters on the CC campus; disseminating recruitment videos targeted to each CC partner, and having CSULB faculty participate in community college events as guest speakers. We are finding that using pictures and quotes from BUILD students who are CC alumni resonates with students on their community college campus. We are working to increase these efforts so CC students are able to picture themselves as the transfer student who is a good fit for CSULB BUILD program activities. These outreach efforts were magnified through the year-long contributions of a VISTA Member (*Volunteers In Service To America* – a domestic Peace Corps type program) whose primary purpose was to support BUILD outreach.

Outcomes of our outreach efforts have been successful overall, but mixed when considering the significantly greater success we have had building on existing relationships than starting new ones. In 2015–16 we conducted more than 50 presentations reaching more than 2000 people (in large part because of our VISTA volunteer). These efforts resulted in a large number of CC student applications to BUILD as shown in Table 2. In our first year, roughly 1/5 of BUILD Scholar applicants and accepted Scholars were CC transfer students. This number increased in year 2 with half of all applicants and a quarter of participating Scholars having spent at least some of their career on a CC campus. While important to have contact with administrators (their buy-in, even if tacit, is helpful), reaching out directly to CC faculty and counselors seems to be more effective. This has not always been easily accomplished as CC department websites are inconsistent and may not include contact information for faculty, coupled with the large number of part-time employees, makes collaborating with CC faculty more problematic on campuses where we did not have a pre-existing relationship. In addition to student outreach, our CC partners have provided us with valuable input regarding our freshmen career exploration course, which is designed to engage students in thinking about different career options, including research. They have offered a course (without the health research focus) similar to this one for years and have good ideas about how to run this type of class. CC faculty have volunteered to work with our faculty to develop and review the class as it is being implemented at CSULB. Given the need and interest in this course at the CC level, BUILD is planning to support a section of this course at our partner campuses. Finally, at the recommendation of our CC partners, we are working to provide them with data about their alumni, which they can use to support requests for funding and program support.

**Table 2:** Community College Alumni Representation in BUILD

	2015–2016 Cohorts			2016–2017 Cohorts		
	# Applications	# Student trainees to join BUILD	Total # BUILD Participants	# Applications	# Student trainees to join BUILD	Total # BUILD Participants *as of 3/3/17
Lower Division BUILD Associates (1-year program)	54	39	39	60*(3 CC)*	35*(2 CC)*	29*(2 CC)*
Upper Division 1 BUILD Scholars (2-year program)	*126(24 CC)*	*47(10 CC)*	*43(9 CC)*	94*(46 CC)*	54*(25 CC)*	41*(11 CC)*
Upper Division 2 BUILD Year 2 Scholars (2-year program)				(N/A – continuing UD)	(N/A – continuing UD)	40

Numbers in parentheses represent community college transfer students who are in their first year at CSULB or previously transferred to CSULB from a community college

*BUILD* Building Infrastructure Leading to Diversity, *CC* community college, *N/A* not applicable, *UD* upper division

#### Research partnerships

Like our successful CC partnership efforts, the research pipeline partnerships developed from existing relationships between faculty on our campus and theirs. This initial entry point for starting and expanding program-wide partnerships cannot be underestimated. It allows us to have a point of contact on the partner campus who provides introductions and immediate buy-in that otherwise takes a long time to develop. We currently have two official research partner institutions for the CSULB BUILD initiative (University of California, Irvine – UCI; University of Southern California – USC) who have committed to engaging and collaborating with BUILD faculty and student trainees on their research through submission of joint grant proposals in research areas of mutual interest (e.g., NIH R01 grants, BRIDGES, IRACDA), a pipeline exchange program where faculty and students from CSULB and research partner institutions can visit research labs on other campuses to enhance their research infrastructure and resources (e.g., USC faculty visiting CSULB), and extending an open invitation to health-related research events and presentations to stimulate the dissemination of research findings (e.g., BUILD colloquium have showcased UCI and USC faculty). In addition, both UCI and USC have committed to participating in the BUILD summer research training program, where upper division BUILD Scholars are paired with a research partner faculty member to assist in ongoing research, become acclimated to the graduate school environment, and enhance networking opportunities for admission to doctoral research programs. To stimulate student interest in applying to UCI and USC summer research training programs, we have coordinated student field trips to these campuses, which has resulted in a number of students being accepted into these summer programs. This collaboration is mutually beneficial as it has the potential to increase the research infrastructure for BUILD faculty mentors, and create sustainable and successful partnerships between campuses that lead to increased opportunities for exceptional student research training and a higher volume of students entering the doctoral pipeline leading to BHS/BSE research careers. Other aspects of this collaboration that have continued to be developed include implementing a pilot post-doctoral scholars program, where underrepresented postdocs would visit CSULB to engage in teaching and research activities, serving as academic role models for URGs. We are also working to provide faculty diversity hiring workshops that are co-developed with our research partners, to strengthen best hiring practices. These partnerships build upon existing collaborations on a larger scale, are sustainable and formalized, and increase capacity to advance students from diverse backgrounds through the doctoral pipeline.

### Evaluation of CSULB BUILD outcomes

Evaluation is a key component to our program goals and activities. A multi-year evaluation plan measures and tracks program-related activities to assess the overarching goals of the CSULB BUILD initiative. The evaluation includes specific plans and activities to assess each of the key intervention levels of the initiative (i.e., Research Infrastructure, Faculty Development, and Student Research Training). The specific aims of the evaluation are to: (1) Implement an evaluation plan to assess both the short- and long-term goals of CSULB BUILD; and (2) Collaborate and coordinate evaluation data collection, activities, and approaches with the Coordination and Evaluation Center (CEC), NRMN and the BUILD Steering committee.

The evaluation of CSULB BUILD is a large-scale project requiring significant resources (e.g., an experienced evaluator, time, data collection and reporting) to carry out the planned activities. Our evaluation team works closely with the CSULB BUILD Leadership team to develop data collection procedures and measures to ensure that project goals, objectives, and processes are met. Data on performance indicators are collected throughout each year to evaluate each BUILD program component. This data-driven approach ensures that changes and improvements to the program can be assessed regularly and implemented by the BUILD Leadership team. The evaluation design utilizes a mixed-methods approach (i.e., both quantitative and qualitative) that includes data collection with all participants (e.g., annual surveys, interviews and focus groups with BUILD students, alumni, and faculty mentors). Other BUILD activities are evaluated using tracking forms, analysis of institutional research data, and conducting interviews with both primary and partner institutional leaders to assess the long-term impact of the CSULB BUILD initiative. The CSULB BUILD evaluation team regularly communicates with the CEC, NRMN, and the BUILD Steering committee and implements their recommendations regarding the use of standardized measures, assessment of Hallmarks of Success, and evaluation activities. The evaluation team is committed to assisting with the consortium-wide evaluation of the BUILD initiative to assess long-term impacts on increasing student, faculty, and institutional participation in BHS/BSE research.

### Institutionalization efforts for sustainability

CSULB has emphasized the importance of institutionalizing the efforts of BUILD. Much of the early work on BUILD was devoted to putting infrastructure in place, developing all the components, and building bridges across the four colleges. Great effort was also made to establish connections across all levels within the university and across divisions (Academic Affairs, Student Services, Administration and Finance, and University Relations and Development) to ensure that all knew about the student, faculty, and institutional components of BUILD and the opportunities for faculty and students. The goal was to establish relationships with people and offices where we could go for help in getting these components up and running and then in institutionalizing them.

People at all levels within CSULB are committed to the success of students and BUILD is an initiative that fits well with our University’s mission. Thus, while there have been many challenges, institutionalization efforts have been supported by campus administrators. Prior to and since receiving funding, both our university President and Provost have been committed to breaking down silos to enhance our research infrastructure; providing university resources to support existing and new programs focused on student and faculty development (including expanding our faculty learning community); developing new courses to recruit, engage, and retain students in BHS/BSE research; facilitating our efforts to strengthen our collaborations with our pipeline and doctoral research partners; and sharing courses with our community college pipeline partners. In addition, they have committed university space for a *Research Success and Student Engagement Center* to facilitate the efforts of our BUILD team, while enhancing student and faculty engagement in BHS/BSE research and fostering interdisciplinary research collaborations. They also committed university funds to develop research facilities and resources to enhance the research infrastructure for faculty and students. Despite the disparate needs of different colleges, the CSULB BUILD initiative has provided a forum for addressing these needs while strengthening cross-college efforts to enhance student success.

We recognized that our state-funded university would not have the resources to keep a complex program like BUILD running in its existing form after NIH funding ended. Thus, our efforts have been, and will continue to be, focused on integrating various components of BUILD into the existing fabric of the institution. The key is to identify offices and centers where BUILD components would naturally fit and enhance their own programmatic efforts and mission. For example, our BUILD Faculty Mentoring Community is something that should be available to all faculty across the university and can easily be run by the Faculty Center for Professional Development. Our BUILD-developed curriculum, websites, and developed resources for student-mentor matching will fit easily into the Office of Undergraduate Research Support (OURS) that was recently launched. Pilot projects, both individual and collaborative, are similar to ones that are sponsored by our Office of Research and Sponsored Programs (ORSP) and, thus, funding will still be available to help increase the research capacity of faculty mentors, making them more competitive for external funding. The collaborative pilot projects will continue to build interdisciplinary research across the colleges. Databases showing available equipment/technologies for research on campus (and at partner institutions) can be maintained by ORSP. Modules that we have developed for training and professional development of our Graduate Assistants are being considered by NRMN to be incorporated into their training materials. Our Graduate Studies Resource Center will provide support for undergraduates applying to doctoral programs and help with GRE preparation. We will continue our pipeline and research partnerships, including offering the course on careers exploration at the community colleges and working with the CSULB Deans and the Vice Provost for Academic Equity, Diversity and Inclusion at UC Irvine in our efforts to increase the diversity of our faculty.

The size of our BUILD training program is large and in a continuing form may need to be smaller with fewer students, but our commitment and mission of preparing students to be highly competitive for doctoral programs in health-related fields will continue. Finding financial support (stipends and hourly wages) for students is one area that will be problematic and may require university-level development efforts, but many of the students in BUILD are eligible for financial aid. Students will be able to enroll in BUILD-developed courses that will count for degree requirements in some departments and will also fulfill general education requirements. Elements of our student research training program will be sustained as we institutionalize the innovative components of the BUILD Learning Community (which can be housed in OURS) to continue supporting students in cultivating a culturally-congruent science identity and sense of belonging to keep them in the BHS/BSE research career pathway. Furthermore, the curriculum for the learning community will be available for dissemination for other institutions as published training manuals. As personal relationships between our campuses and individual faculty develop and thrive, so too will the institutional partnerships between CSULB and our pipeline and research partners, allowing cross campus opportunities for research and growth.

## Additional files

Additional file 1: Selected list of past and current student research training programs at California State University, Long Beach (CSULB). This file includes a selected list of past and current student research training programs at CSULB by program name, dates of operation, program methods and objectives, and key findings and results. (DOCX 22 kb)

Additional file 2: Overview of student learning goals and skill development from research curriculum at California State University, Long Beach (CSULB). This file includes an overview of the student learning goals for each of the courses developed to be part of the BUILD (and campus) research curriculum. (DOCX 22 kb)
