# Supplementary material for: Advancing research opportunities and promoting pathways in graduate education: a systemic approach to BUILD training at California State University, Long Beach (CSULB)
Source: BMC Proc. 2017 Dec 4;11(Suppl 12):26. doi: 10.1186/s12919-017-0088-3 (PMC5773868; doi:10.1186/s12919-017-0088-3)
Supplement: Supplementary file 1 — Selected list of past and current student research training programs at California State University, Long Beach (CSULB). This file includes a selected list of past and current student research training programs at CSULB by program name, dates of operation, program methods and objectives, and key findings and results. (DOCX 22 kb) [file 12919_2017_88_MOESM1_ESM.docx]

**Additional File 1**

Additional File 1: Selected list of past and current student research training programs at California State University, Long Beach (CSULB) including the name of the program, dates of operation, methods and objectives, and key findings and results.

**Legend:** CBPR = community-based participatory research; CBRS = Center for Behavioral Research and Services; CC = community college; CHER = Center for Health Equity Research; CHHS = College of Health and Human Services; CLA = College of Liberal Arts; CNSM = College of Natural Sciences & Mathematics; COE = College of Engineering; COR = Career Opportunities in Research Program; GPA = grade point average; H2OLA = Hispanic Health Opportunity Learning Alliance; HSI = Hispanic Serving Institution; LSAMP = Louis Stokes Alliance for Minority Participation Program; MARC U*STAR = Maximizing Access to Research Careers Undergraduate Student Training in Academic Research; MCH = maternal and child health; MRISP = Minority Research Infrastructure Support Program; NCLR = National Center of La Raza; NIGMS = National Institute of General Medical Sciences; NIH = National Institutes of Health; NIMH = National Institute of Mental Health; NIMHD = National Institute on Minority Health and Health Disparities; NSF = National Science Foundation; RIMI = Research Infrastructure in Minority Institutions; RISE = Research Initiative for Scientific Enhancement; STEM = Science, Technology, Engineering, and Mathematics; UG = undergraduate; UR = under represented; URS = underrepresented students; USDA = United States Department of Agriculture

| **METHODS & OBJECTIVES** | **KEY FINDINGS & RESULTS** |
| --- | --- |
| **Past Programs** | |
| **NIMH COR; Psychology Department CLA (1981-2012)** | |
| - 2-year research training for UR undergraduates - Summer program with doctoral institution - Develop research with faculty mentor | - 94% of students completed 2-year program - 89% entered graduate school (53% in doctorate) - 67% entering doctorate completed degree |
| **Latino Healthcare Professionals Project; CHHS/NCLR (1995-2010)** | |
| - Mentoring to 193 1st-generation educated UG - ↑ graduate school admission/completion - ↑ workforce representation | - 93% program completion (65% honors lists) - 67% attended graduate school within 5 years - 76% worked full time in health-related careers |
| **US Department of Education: *Mi Casa: Mi Universidad*; CHHS/NCLR (2005-2011)** | |
| - Improve retention & graduation rates - 65 Latino peer mentors trained - Culturally relevant pedagogy & family events | - Mentoring to 401 CNSM, CHHS, & COE URS peers - Statistically significant ↑ in mentee GPA - All incoming faculty trained in CBPR |
| **NIMH MRISP; Psychology Department CLA (2006-2010)** | |
| - 10 junior faculty trainees - 4-year research training in health disparities - Training & mentoring with R1 partners | - 89 conference presentations (70% with students) - 30 publications (28% with students) and 2 funded grants - Hosted regional conference on mental health disparities |
| **USDA: *Comienzo Sano I & II*; CHHS/NCLR (2007-2011)** | |
| - 1-year CBPR to improve Latino MCH - Train 32 1^st^ gen-educated in CBPR (2 grads) - Course-Latino Nutrition & Disease Prevention | - 100% completed bachelors (5 masters, 1 doctoral) - ↑ knowledge of research & graduate school prep - ↑ research skills & experience with faculty mentor |
| **NIMHD RIMI; CHER, CBRS, & NCLR/CSULB Center (2009-2014)** | |
| - 1-year graduate health disparities research w/faculty - Course-Health Disparities Research | - 100% of students completed the 1-year program - 50% entered doctoral programs within two years |
| **USDA *Sanos y Fuertes*; CHHS/NCLR (2010-2015)** | |
| - 35 bilingual/bicultural grads trained in CBPR - 375 Latino families in obesity prev. research - Latino Health/Nutrition Graduate Program | - ***Centro Salud es Cultura*** opened 10/2013 - 297 families intervention to-date (96.6% retention) - Significant results p. <.001 for all major objectives |
| **Current Programs** | |
| **NIH Bridges to the Baccalaureate; CNSM (1990s-present)** | |
| - UR CC students participating in faculty-directed biomedical research - Advising transfer & grad requirements | - > 90% transferred to 4-year (50% CSULB) - Many continue in faculty research; 95% graduate - > 80% of alumni graduate w/in 3 years after transfer |
| **NSF LSAMP; CNSM (1990s-present)** | |
| - ↑ URS participation in research since 1990s - ↑ URS students transitioning to MS & PhD - Insuring navigation through the pipeline | - > 800 STEM and non-STEM students have participated - > 80% complete undergraduate degrees - > 50% continue on to graduate work |
| **MARC U*STAR; CNSM & CLA (2000-2017)** | |
| - 2-year research training & faculty mentorship - Summer program in research institutions - Develop academic, research, and leadership skills | - 100% presented at national conferences/publications - 86% in 10-week summer training at R1 campus - 65% entered graduate research programs |
| **NIGMS RISE; CNSM (2005-2019)** | |
| - UR undergraduates in biomedical sciences - 2-year research training - Develop research with faculty mentor | - 83% completed 2-year Fellows Program - 61% in graduate or professional schools - 51% Biomedical MS or PhD programs |
| **US Department of Education: HSI STEM; CNSM, COE, & NCLR (2011-2016)** | |
| - 20 CNSM & COE Latino peer mentors/year - Improve STEM retention & graduation rates - Summer/Winter faculty-student research | - Provided tutoring to 781 students (sig ↑ in GPA) - 97 Latino mentees in weekly meetings - 70 students in Summer & Winter Research Program |
| **NIMHD H_2_OLA; CHHS/NCLR & CNSM (2011-2016)** | |
| - 7 Latino peer mentors/year & 35 mentees - Improve biomedical retention & graduation rates - ↑ involvement in Hispanic health equity research | - Provided mentoring to 82 students - 73 Latino student research posters; 83% ↑ research skills - Over 1000 attendees at Latino Health Equity Conference |
