# Supplementary material for: Advancing research opportunities and promoting pathways in graduate education: a systemic approach to BUILD training at California State University, Long Beach (CSULB)
Source: BMC Proc. 2017 Dec 4;11(Suppl 12):26. doi: 10.1186/s12919-017-0088-3 (PMC5773868; doi:10.1186/s12919-017-0088-3)
Supplement: Supplementary file 2 — Overview of student learning goals and skill development from research curriculum at California State University, Long Beach (CSULB). This file includes an overview of the student learning goals for each of the courses developed to be part of the BUILD (and campus) research curriculum. (DOCX 22 kb) [file 12919_2017_88_MOESM2_ESM.docx]

**Additional File 2**

Additional File 2: Research Curriculum Skills Table used to identify topics to be covered in Research Curriculum Courses and which course would have primary responsibility, and which courses would support the topic by providing an initial introduction or expansion by revisiting the topic in a later course.

**Legend:** CE = Career Exploration; HD = Health Disparities; IRM = Introduction to Research Methods; SRC = Scientific Research Communication; ARM for Advanced Research Methods; and ALL if it should be evenly spread over all courses.

**Topics incorporated into All Courses**

| Ability to work effectively as part of a group | All |
| --- | --- |
| Team working skills/synergistic activities. | All |

**Topics Primarily in Career Exploration Supporting**

| Prepare a professional development plan (PDP). | All |
| --- | --- |
| Describe the process of becoming a professional scientist beginning with the undergraduate studies, to graduate school, postdoctoral studies and the professional career (professor, research scientist, etc). |  |
| Discuss the lifestyle of a PhD student in biomedical or behavioral sciences. |  |
| Curiosity/creativity | All |
| Mental/emotional toughness (grit, persistence, resiliency) *Growth Mindset* Strong academic background (not peaked yet) | All |
| Time management/Stress management |  |

**Topics Primarily in Interdisciplinary Approaches to Health Disparities**

| Articulate what is meant by diversity and its value in science. | All |
| --- | --- |

**Topics Primarily in Introduction to Research Methods Primarily Supporting**

| Time Management/Project Management - Instill in students that they are responsible for their research project | All |
| --- | --- |
| Understand accepted norms for ethical research conduct and reporting, ethics and knowledge on human/animal subject training, Articulate the importance of ethical conduct in the practice and reporting of research. | HD |
| Understand the essential process of doing science (Steps in a research study) | ARM |
| The logic of experimental design, very basic ideas about reliability and validity |  |
| Describe the fundamental components of executing a publishable experiment (e.g. treatments, controls, protocol). | ARM |
| Identify type of analysis to answer a research question (Also primary in Advanced Research Methods) |  |
| Being able to provide rationales for the proposed hypotheses (Also primary in Advanced Research Methods) |  |
| Basic knowledge of survey development (Behavioral) | ARM |
| How to read and understand a very basic scatter plot with X, Y axis | SRC,ARM |
| Be able to perform, interpret, and present (tables/figures) basic descriptives and simple analyses (ttest, correlation) | SRC |
| Understanding the difference between experimental and control data sets | ARM |
| The concept of confounding in observational or other non-experimental designs |  |
| Difference between IV, DV | ARM |

**Topics Primarily in Scientific Research Communication Supporting**

| Understand the direct connection between clear writing and clear thinking (importance of writing throughout the research process). Be able to write clear concise English sentences using accepted grammar and syntax. | All |
| --- | --- |
| Be able to read and comprehend complex arguments. | ARM |
| Conduct a lit review and properly cite | IRM, ARM |
| Contrast peer-reviewed, non-peer-reviewed and layperson research articles. | CE IRM |
| Be to write effectively for different audiences. | ARM |
| Technical writing | ARM |
| Be able to craft a technical report presenting research data clearly and succinctly. | IRM, ARM |
| Be able to present orally scientific information in a clear concise manner. | IRM, ARM |
| Prepare a poster for presenting results at a scientific meeting. | ARM |
| Prepare an oral presentation of research results that could be delivered at a scientific meeting. | ARM |
| Write a simple research proposal/prospectus | IRM, ARM |

**Topics Primarily in Advanced Research Methods Supporting**

| Understand what problems are worth tackling | IRM,HD,SRC |
| --- | --- |
| Define a hypothesis as it relates to biomedical and behavioral research. | IRM, SRC |
| Describe how scientific literature is used to construct hypotheses. | IRM, SRC |
| Develop research questions and hypotheses | IRM |
| Being able to provide rationale for the proposed hypotheses | IRM |
| Plan a preliminary study that is based upon the primary scientific literature to test a hypothesis. |  |
| How to critically evaluate past studies, How to look for the limitations of past studies |  |
| Identify type of analysis to answer a research question | IRM |
| Analyze and interpret the results of a preliminary study with the mindset of planning a complete experiment. |  |
| Understand the differences among various research designs and the typical methods employed in each design |  |
| Describe fundamental/cutting-edge research methods used in biomedical or behavioral research | IRM, HD |
| Be able to perform, interpret, and present (tables/figures) basic descriptives and simple analyses (ttest, correlation) | SRC |
| What an interaction is (Behavioral) |  |
| Applied data analysis | IRM |
| Computer programming (e.g. Matlab) (Biomedical) |  |
| Peer-reviewed journals and how a manuscript is reviewed by the journal editor and by peers. | SRC |

**Topics Primarily in Subject for Proposed Application Preparation Course**

| Engage in a mock-interview for an on-campus interview for a PhD program. |
| --- |
| Describe the importance of each component of an application to a PhD program in biomedical or behavioral sciences. |
| Prepare a timeline for preparing and assembling the materials needed for an application to biomedical and behavioral PhD programs. |
| Identify the range of PhD programs that support biomedical and behavioral research. |
| Prepare applications to PhD programs in biomedical or behavioral sciences. |
| Describe the anticipated experience and expectations of an on-campus interview for gaining admission to a PhD program. |
| Engage in a mock-interview for an on-campus interview for a PhD program. |
| Describe the importance of each component of an application to a PhD program in biomedical or behavioral sciences. |
